# The significance of *PIWI* family expression in human lung embryogenesis and non-small cell lung cancer

**DOI:** 10.18632/oncotarget.3003

**Published:** 2015-01-23

**Authors:** Alfons Navarro, Rut Tejero, Nuria Viñolas, Anna Cordeiro, Ramon M. Marrades, Dolors Fuster, Oriol Caritg, Jorge Moises, Carmen Muñoz, Laureano Molins, Josep Ramirez, Mariano Monzo

**Affiliations:** ^1^ Molecular Oncology and Embryology Laboratory, Human Anatomy Unit, School of Medicine, University of Barcelona, IDIBAPS, Barcelona, Spain; ^2^ Department of Medical Oncology, Institut Clinic Malalties Hemato-Oncològiques (ICMHO), Hospital Clinic de Barcelona, University of Barcelona, IDIBAPS, Barcelona, Spain; ^3^ Department of Pneumology, Institut Clínic del Tórax (ICT), Hospital Clinic de Barcelona, University of Barcelona, IDIBAPS, CIBER de Enfermedades Respiratorias (CIBERES), Barcelona, Spain; ^4^ Department of Pathology, Centro de Diagnóstico Biomédico (CDB), Hospital Clinic de Barcelona, University of Barcelona, IDIBAPS, CIBERES, Barcelona, Spain; ^5^ Department of Thoracic Surgery, Institut Clínic del Tórax (ICT), Hospital Clinic de Barcelona, University of Barcelona, Barcelona, Spain

**Keywords:** PIWI proteins, piwiRNAs, PIWIL1, PIWIL4, NSCLC

## Abstract

The expression of Piwi-interacting RNAs, small RNAs that bind to PIWI proteins, was until recently believed to be limited to germinal stem cells. We have studied the expression of *PIWI* genes during human lung embryogenesis and in paired tumor and normal tissue prospectively collected from 71 resected non-small-cell lung cancer patients. The mRNA expression analysis showed that *PIWIL1* was highly expressed in 7-week embryos and downregulated during the subsequent weeks of development. *PIWIL1* was expressed in 11 of the tumor samples but in none of the normal tissue samples. These results were validated by immunohistochemistry, showing faint cytoplasmic reactivity in the *PIWIL1*-positive samples. Interestingly, the patients expressing *PIWIL1* had a shorter time to relapse (TTR) (*p* = 0.006) and overall survival (OS) (*p* = 0.0076) than those without *PIWIL1* expression. *PIWIL2* and *4* were downregulated in tumor tissue in comparison to the normal tissue (*p* < 0.001) and the patients with lower levels of *PIWIL4* had shorter TTR (*p* = 0.048) and OS (*p* = 0.033). In the multivariate analysis, *PIWIL1* expression emerged as an independent prognostic marker. Using 5-Aza-dC treatment and bisulfite sequencing, we observed that *PIWIL1* expression could be regulated in part by methylation. Finally, an *in silico* study identified a stem-cell expression signature associated with *PIWIL1* expression.

## INTRODUCTION

Non-small-cell lung cancer (NSCLC) accounts for 85% of all lung cancers and has a 5-year survival rate of 16% [1]. Surgery is the initial treatment for early-stage NSCLC patients, but even after complete resection, recurrence rates are substantial (20–85%, depending on tumor stage) [2], highlighting the need for prognostic markers. From a stem-cell point of view, a tumor can be viewed as an aberrant organ initiated by a tumorigenic cancer cell that acquires the capacity for indefinite proliferation through accumulated mutations [3], indicating that the study of embryogenesis can be a good source of prognostic markers. Several studies have shown that cancer recapitulates the gene expression pattern found in the early development of the corresponding organ [4–7] and genes that are functionally altered in lung cancer, including non-coding RNA genes, play a key role in lung development [8].

The recognition of the importance of non-coding RNAs in cancer biology has increased considerably in recent years, with a special focus on small non-coding RNAs (<200bp long) [9]. One of the most frequent functions of small non-coding RNAs is the silencing of RNA expression by a base pairing interaction mechanism [10], which always involves a member of the Argonaute family that binds to the small non-coding RNAs. The PIWI protein subfamily of Argonaute proteins is composed of four members in humans: PIWIL1 (HIWI), PIWIL2 (HILI), PIWIL3 and PIWIL4 (HIWI2) [11]. The PIWI proteins bind to Piwi-interacting RNAs (piRNAs) [12], which are small, 24–32nt long, single-stranded RNAs. Until recently, piRNAs were believed to be expressed exclusively in the germ line [13, 14], but they are now known to be more universally expressed and have been detected in somatic and tumor cells [15–17]. Multiple functions have been associated with the PIWI/piRNA pathway, including the repression of transposons, which can multiply and move to new positions in the genome. By repressing transposons, the PIWI/piRNA pathway thus procures the genomic stability of germline cells [18]. piRNAs also target mRNAs, leading to their degradation, and are involved in epigenetic regulation in the nucleus of the cell [19, 20].

piRNA biogenesis is Dicer-independent and mediated by PIWI proteins [21]. Two main pathways of piRNA biogenesis have been described: the primary pathway and the secondary pathway (also known as the “ping-pong” amplification cycle). Little is known about the molecules that participate in piRNA biogenesis in humans, but PIWIL1 is known to act exclusively in the primary pathway and PIWIL4 in the secondary pathway, while PIWIL2 acts in both [14]. The primary pathway is believed to produce new piRNAs, while the secondary pathway is believed to be in charge of maintaining the total pool of active piRNAs in the cell [22–24].

In several cancer cell lines, *PIWIL1* overexpression has been related to cell proliferation [25–27] and *PIWIL2* overexpression to anti-apoptotic signaling and cell proliferation [28, 29]. Interestingly, *PIWI* genes are associated with stem cell self-renewal and are re-expressed in precancerous stem cells that have the potential for malignant differentiation [30, 31]. The first report of *PIWI* expression in tumor tissue was in seminomas, where *PIWIL1* was detected in the tumor but not in normal tissue [22]. *PIWIL1* overexpression has since been detected in sarcomas, where it drives tumorogenesis via increased global DNA methylation [32], in colorectal cancer [33], where it plays a role as a prognostic marker, and in other tumors (reviewed in [34]). However, the role of *PIWI* genes in NSCLC has not been studied.

**Figure 1:**
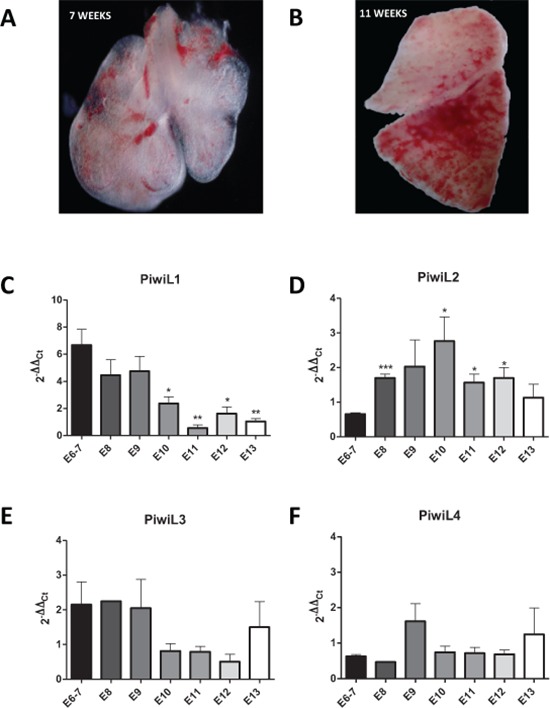
Images of the embryonic lung at **(A)** 7 weeks and **(B)** 13 weeks Expression levels of **(C)** *PIWIL1*, **(D)** *PIWIL2*, **(E)** *PIWIL3*, and **(F)** *PIWIL4* in the lung from week 6 to 13 of embryonic development.

We hypothesized that the PIWI/piRNA pathway may be an embryonic mechanism that is inactivated in adult differentiated lung cells and reactivated during tumorogenesis. To clarify this role of *PIWI* genes, we have examined their expression in human embryonic lungs (Figure 1A and 1B) and in paired tumor and normal tissue from surgically resected NSCLC patients and correlated our findings with patient outcome.

## RESULTS

### *PIWI* family expression during human lung embryogenesis

The expression of the *PIWI* genes *PIWIL1–4* was assessed in human embryonic lung tissue from embryos of 6–13 weeks of development (Figure 1C–1F). The median 2^−ΔCt^ values were 0.0084 and 0.0055 for *PIWIL4* and *PIWIL2*, respectively, in comparison with 0.00014 and 0.000057 for *PIWIL1* and *PIWIL3*, respectively. *PIWIL4* thus had the highest expression levels. When we compared embryonic *PIWI* expression longitudinally from 6 to 13 weeks, significant changes in expression were observed only in *PIWIL1* and *PIWIL2* levels as the lung became more differentiated. *PIWIL1* showed a significant reduction in overall levels from week 6 to 13 (Figure 1C), while *PIWIL2* showed a curved expression with an increase beginning at week 8, peaking at week 10, and decreasing to initial levels at week 13 (Figure 1D).

### *PIWI* family expression in tumor and normal tissue samples and correlation with clinical characteristics

**Figure 2:**
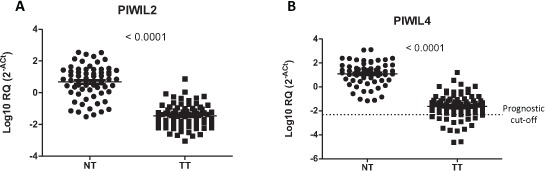
Expression of **(A)** *PIWIL2* and **(B)** *PIWIL4* in paired normal (NT) and tumor tissue (TT) from patients with non-small-cell lung cancer.

Seventy-one patients were included in the analysis. All patients had pathologically confirmed stage I–III NSCLC and Eastern Cooperative Oncology Group performance status 0–1 (Table 1). In tumor tissue, *PIWIL1* expression was detected in eleven patients (15.5%), *PIWIL2* in 66 (93%), *PIWIL3* in five (7%) and *PIWIL4* in 60 (84.5%). In normal tissue, only *PIWIL2* and *PIWIL4* were expressed, while *PIWIL1* and *PIWIL3* were not detected in any of the 71 normal tissues examined. Both *PIWIL2* (Figure 2A) and *PIWIL4* (Figure 2B) were significantly downregulated in tumor tissue compared to normal tissue (*p* < 0.001).

**Table 1:** Patient characteristics

Characteristics	Value	*N* = 71 N (%)	*p*-value*	
			TTR	OS
Sex	Male	60 (84.5)		
	Female	11 (15.5)	0.099	0.059
Age, yrs.	Median (Range)	68 (46–83)		
	< = 65	31 (43.7)		
	> 65	40 (56.3)	0.183	0.122
ECOG PS	0	7 (9.9)		
	1	64 (90.1)	0.971	0.231
Stage	I	44 (62)		
	II	17 (23.9)		
	III	10 (14.1)	0.055	0.008
Histology	Adenocarcinoma	40 (56.3)		
	Squamous cell carcinoma	31 (43.7)	0.845	0.313
Type of surgery	Lobectomy/Bilobectomy	60 (84.5)		
	Pneumonectomy	4 (5.6)		
	Atypical resection	7 (9.9)	0.830	0.491
Smoking history	Current smoker	24 (33.8)		
	Former smoker	41 (57.7)		
	Never smoker	4 (5.6)		
	Unknown	2 (2.8)	0.315	0.109
Adjuvant treatment	Yes	19 (26.8)		
	No	52 (73.2)	0.738	0.740
Relapse	No	45 (63.4)		
	Yes	26 (36.6)		
p53 mutations	Yes	13 (18.3)		
	No	55 (77.5)		
	Unknown	3 (4.2)	0.181	0.905
K-ras mutations	Yes	10 (14.1)		
	No	56 (78.9)		
	Unknown	5 (7)	0.533	0.391

* log-rank *p*-value for comparison of groups in univariate analysis.

TTR, time to relapse; OS, overall survival.

*PIWIL2* and *PIWIL4* were downregulated in current and former smokers compared to never smokers (* p* = 0.01 and *p* = 0.003, respectively). *PIWIL2* was also downregulated in patients with lymph node involvement (N0 vs N1/2; *p* = 0.038). No other correlation with clinical or molecular characteristics and *PIWIL1–4* expression was observed.

### *PIWIL1* and *PIWIL4* expression and clinical outcome

**Figure 3:**
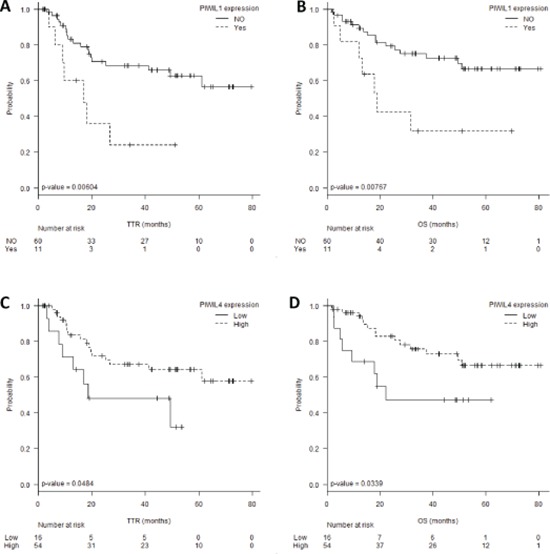
**(A)** Time to relapse (TTR) and **(B)** overall survival (OS) according to *PIWIL1* expression levels. **(C)**TTR and **(D)** OS according to *PIWIL4* expression levels.

The 11 patients expressing *PIWIL1* (*PIWIL1* Ct < 35 and *β-actin* < 35) had shorter TTR (22 vs 55 months; *p* = 0.006) and OS (32 vs 61 months; *p* = 0.0076) than those not expressing *PIWIL1* (Figure 3A–3B). Using the cut-off identified by the Maxstat package of R [35], we observed that patients with lower levels of *PIWIL4 (PIWIL4* log10 [2^−ΔCt^] ≤–2.31, percentile 21.5%, Figure 2) had shorter TTR (30 vs 56 months; *p* = 0.048) and OS (36 vs 62 months; *p* = 0.033) than those with high levels (Figure 3C–3D).

In the multivariate analyses, *PIWIL1* expression emerged as an independent prognostic factor for TTR (OR, 2.892, 95% CI: 1.132–7.388; *p* = 0.026) and OS (OR, 2.833, 95% CI: 1.044–7.685; *p* = 0.041) (Table 2).

**Table 2:** Multivariate analyses

Time to Relapse	Odds Ratio (95% CI)	*p*-value
Male sex	—	0.119
Stage I	—	0.252
*PIWIL1* expression	2.892 (1.132–7.388)	0.026
Low *PIWIL4* expression levels	—	0.089
**Overall Survival**	**Odds Ratio (95% CI)**	***p*-value**
Male sex	6.668 (1.384–32.116)	0.018
Stage I	0.270 (0.091–0.802)	0.018
*PIWIL1* expression	2.833 (1.044–7.685)	0.041
Low *PIWIL4* expression levels	—	0.220

### PIWIL1 analysis by immunohistochemistry

**Figure 4:**
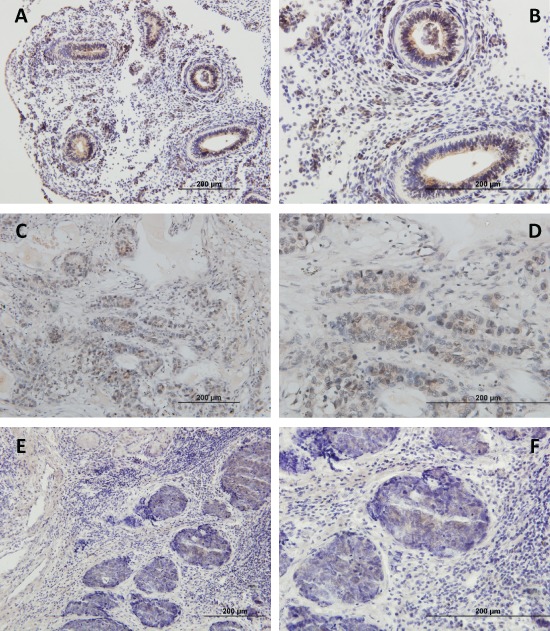
Immunohistochemical analysis of PIWIL1 expression in **(A, B)** embryonic lung tissue, **(C, D)** tumor tissue from a patient with squamous cell lung cancer, **(E, F)** tumor tissue from a patient with lung adenocarcinoma. The images in the right column are a magnification of the images in the left column.

The study of *PIWIL1* expression was validated at protein level by immunohistochemistry in 20 NSCLC cases (10 *PIWIL1*-positive and 10 *PIWIL1*-negative). The *PIWIL1*-positive cases showed faint cytoplasmic reactivity (Figure 4). Interestingly, the study by immunohistochemistry of embryonic samples showed that the expression of PIWIL1 was mainly found in the epithelial compartment of the embryonic lung (Figure 4A and 4B). Figure 4C and 4D show PIWIL1 expression in tumor tissue from a patient with squamous cell lung cancer. Figure 4E and 4F show PIWIL1 expression in a tumor tissue from a patient with lung adenocarcinoma.

### *PIWI* family gene expression is partially regulated by methylation and correlates with overall methylation levels

**Figure 5:**
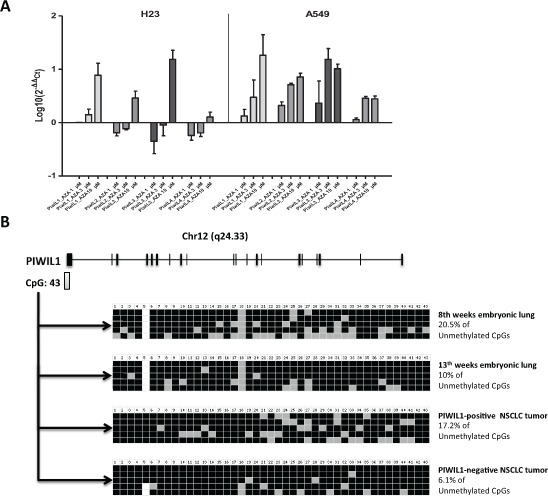
Study of the methylation of *PIWI* genes in lung cancer **(A)** 5-Aza-dC treatment of H23 and A549 lung cancer cell lines showed an increase of the expression levels of the four *PIWI* genes. **(B)** Schematic representation of the *PIWIL1* gene showing the presence of a CpG island close to the first exon of *PIWIL1* gene. The bisulfite sequencing analysis of the 43 CpG island showed differences between a *PIWIL1*-positive and a *PIWIL1*-negative case.

Since *PIWI* genes had previously been described as regulated by methylation in human seminomas [36], we hypothesized that they could also be regulated by methylation in NSCLC. We treated two NSCLC cell lines A549 and H23 with the demethylating agent 5-Aza-dC and analyzed the expression of the four *PIWI* genes. A dose-dependent increase in the expression levels of the four genes after 5-Aza-dC treatment was observed (Figure 5A). We then focused further analyses on *PIWIL1*. We performed bisulfite sequencing analysis of the 43 CpG island located in its promoter region in 4 samples (one 8-week embryonic lung, one 13-week embryonic lung, one *PIWIL1*-positive tumor and one *PIWIL1*-negative tumor) (Figure 5B). The 8-week embryonic lung showed the highest unmethylation level (20.5%). The *PIWIL1*-positive case showed higher unmethylation (17.2%) than the *PIWIL1*-negative case (6.1%) (Figure 5B).

Finally, since epigenetic functions have been associated with piRNAs, we examined the relation between the expression of the *PIWI* genes and overall changes in methylation levels in tumor samples. We observed a positive correlation of *PIWIL4* levels with overall methylation (*r* = 0.267, *p* = 0.044).

### Enriched gene signatures associated with *PIWIL1* expression

**Figure 6:**
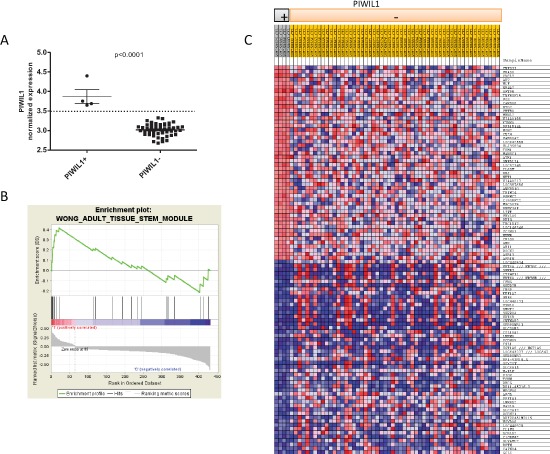
*In silico* analysis using GEO public data (GSE10245) to identify gene expression signatures associated with *PIWIL1* expression **(A)** Classification of patients according to *PIWIL1* expression. Four patients were classified as *PIWIL1*-positive. **(B)** Enrichment plot showing that the gene expression signature associated with *PIWIL1*-positive patients is enriched in the Wong_Adult_Tissue_Stem_Module. **(C)** Heat map of genes used by GSEA after filtering to perform the analysis. The heat map shows significant differences in the gene expression signature between *PIWIL1*-positive and *PIWIL1*-negative patients.

Since *PIWIL1* was highly expressed during early lung development and *PIWIL1* expression in tumor tissue was a marker of poor prognosis, we postulated that its expression could be associated with stem-cell characteristics. We therefore compared *in silico* the expression patterns associated with *PIWIL1*-positive patients vs *PIWIL1*-negative patients. Using the public data available in GEO, we used the data from expression arrays performed in 58 NSCLC cases (GSE10245; PMID: 18486272). In these patients, we studied the expression of *PIWIL1* gene and identified four patients expressing *PIWIL1* (Figure 6A). We then compared gene expression between these four patients and the 54 *PIWIL1*-negative patients and were able to identify a 451-gene signature (72 upregulated and 379 downregulated) associated with *PIWIL1* expression (Supplementary Table 1). To study the functional relevance of this signature, we performed a Gene Set Enrichment Analysis (GSEA). GSEA identified several gene signatures (Supplementary Figure 1), including a gene signature associated with stem cells (“Wong_Adult_Tissue_Stem_Module”) (Figure 6B) [37]. Figure 6C displays all the genes included in the GSEA analysis after the filtering performed by the program; significant differences were observed between the *PIWIL1*-positive and *PIWIL1*-negative group.

## DISCUSSION

In the present study, we have examined whether the re-activation of the PIWI/piRNA pathway in tumor tissue influences patient outcome in NSCLC. The PIWI/piRNA pathway was until recently thought to be active exclusively in germinal cells and in the first stages of embryonic development [14]. However, we have shown that *PIWI* expression can be detected during lung organogenesis, with a distinct expression pattern for each *PIWI* gene. Moreover, some *PIWI* genes can be detected in tumor and normal tissue – again with distinct expression patterns.

In piRNA biogenesis, we can distinguish two main pathways: the primary pathway and the secondary pathway. *PIWIL1* participates exclusively in the primary pathway. The expression pattern of *PIWIL1* indicates that while the primary pathway is very active during the early stages of lung embryogenesis, this pathway becomes downregulated during the differentiation of the embryonic and fetal lung and is finally inactivated in normal adult lung tissue. Interestingly, this pathway is reactivated in some patients, where it is a marker of poor prognosis. It has been shown in seminoma patients that this inactivation may be mediated by methylation [36]. To corroborate if this methylation-mediated inactivation would hold true in NSCLC, we treated two NSCLC cell lines with 5-Aza-dC, which led to the re-expression of all *PIWI* family members. Moreover, we analyzed a *PIWIL1*-positive and a *PIWIL1*-negative tumor sample by bisulfite sequencing and found differences in the degree of methylation between the two samples. These results lead us to speculate that in lung cancer, *PIWIL1* inactivation may well be mediated in part by methylation of its promoter region. Other mechanisms have also been shown to affect *PIWIL1* expression. Reeves and coworkers found that *PIWIL1* expression was higher in NSCLC cell lines (A549 and NCI-H1299) than in normal lung epithelial cells (CRL-9482) and that the expression of *PIWIL1* could be activated by the oncogene RASSF1C [38].

In the present study, we have shown that the re-activation of *PIWIL1* in tumors is associated with a stem-cell expression signature. Liang *et al*. [39] have recently reported that *PIWIL1* is essential to the maintenance of lung cancer stem cell populations. Knockdown of *PIWIL1* compromised sphere formation ability in the stem cell population SSC^lo^ Alde^br^ and *in vivo* tumor growth in a nude mice model [39]. In a previous study, the same group showed that *PIWIL1* gene silencing decreased proliferation and promoted apoptosis in lung cancer stem cells [40]. Although the prognostic significance of *PIWIL1* expression has not previously been examined in NSCLC, it has been associated with poor outcome in other tumors, including glioma [26], pancreatic cancer [41], colorectal cancer [42], esophageal cancer [43], liver cancer [44], gastric cancer [45], and sarcomas [25, 46]. Furthermore, in glioma [47], breast cancer [48] and sarcomas [49], *in vitro* studies have shown that the suppression of *PIWIL1* caused inhibition of cell growth by different mechanisms. Our finding that *PIWIL1* expression was related to shorter TTR (*p* = 0.006) and OS (* p* = 0.0076) is along the same lines as these studies in other tumors and indicate that *PIWIL1* re-expression could be a useful prognostic and therapeutic marker. Moreover, PIWIL1 protein levels can be easily detected in the pathology laboratory by immunohistochemistry.

*PIWIL2* and *PIWIL4* are active in the secondary pathway of piRNA biogenesis, where they participate in the “ping-pong” amplification cycle. *PIWIL2* and *PIWIL4* were both expressed in all samples – both in the embryonic and the adult normal and tumor tissue. In fact, during lung organogenesis (6–13 weeks), *PIWIL2* and *PIWIL4* expression is higher than that of *PIWIL1* or *PIWIL3*. *PIWIL4* expression remained high throughout the 6–13 weeks of development. In contrast, *PIWIL2* expression increased significantly during the transition from the embryonic to the pseudoglandular stage of lung development, when there is a high rate of cell proliferation in order to produce the branching of the fetal lung. At embryonic weeks 6–8, endodermic buds appear, surrounded by mesenchymal mesodermic cells. At week 10, the endodermic cells acquire the characteristics of pseudostratified epithelium, and branching occurs. At week 12, branching morphogenesis continues and the bronchial lumen can be observed, covered by a cubic endodermic epithelium [7]. The changes observed in *PIWIL2* expression and the consistently high expression of *PIWIL4* during this time could be related to this high rate of proliferation, highlighting the importance of the secondary piRNA pathway during this transition phase. Both *PIWIL2* and *PIWIL4* were also expressed in tumor and normal tissue, indicating that the secondary pathway is not inactivated in adult tissue. Interestingly, *PIWIL2* and *PIWIL4* were expressed at significantly lower levels in tumor than in paired normal tissue (*p* < 0.001). Importantly, patients with lower levels of *PIWIL4* had shorter TTR (*p* = 0.048) and OS (*p* = 0.033).

It has been postulated that the secondary pathway is in charge of the maintenance of the pool of piRNAs of the cell, especially those targeting transposons [14]. This would match with the fact that *PIWIL2* and *PIWIL4* were detected in all our samples. Moreover, the downregulation of these genes in tumor samples could be related to an increase of transposon activities, resulting in genomic instability [50]. One of the mechanisms used by the piRNAs to silence transposons is DNA methylation [51]. It has been shown that PIWIL4 can modulate chromatin modifications through methylation of H3K9, specifically in the p16Ink4a locus [52]. In our study, we have observed a positive correlation between *PIWIL4* levels and overall methylation. However, since the overall methylation levels were quantified using an ELISA method, it is not clear if the overall methylation corresponds to transposon regions or other transposable elements regulated by piRNAs. This interesting question warrants further study with more complex techniques.

In summary, we have examined the expression of *PIWI* genes in order to determine the activity and potential prognostic role of the PIWI/piRNA pathway in NSCLC. *PIWIL1* participates in the primary pathway and *PIWIL2* and *PIWIL4* in the secondary pathway, both of which are active in NSCLC. The re-expression of the *PIWIL1* gene, which can be confirmed by immunohistochemistry, is related to poor prognosis and is associated with a stem-cell signature. The downregulation of *PIWIL4* is also related to poor prognosis and is associated with lower methylation. Further investigation in a larger cohort of patients is warranted to validate these findings and to examine potential diagnostic and therapeutic approaches.

## MATERIALS AND METHODS

### Study population

Seventy-one fresh tumor samples and 71 paired normal tissue samples from NSCLC patients who underwent complete surgical resection in our institution were prospectively collected between June 2007 and March 2011. Immediately after surgery, samples were labelled, frozen at −80°C and kept for further processing. Approval for the study was obtained from the Institutional Review Board of the Hospital Clinic of Barcelona, Spain. Written informed consent was obtained from each participant in accordance with the Declaration of Helsinki.

Twenty clinically aborted embryos and fetuses were donated with written informed consent to the Body Donation Service of the Human Anatomy and Embryology Department of the School of Medicine of University of Barcelona for morphological and molecular studies. The samples included lungs from the 6th to the 13th week of development. Lung samples were obtained under control of an Olympus stereo microscope SZ61. Samples for RNA extraction were frozen in liquid nitrogen and stored at −80°C until analyzed.

### RNA extraction and mRNA quantification

Total RNA was extracted from tumor and normal frozen tissue using Trizol (Life Technologies, Foster City, CA) according to the manufacturer's protocol. RNA from samples was quantified using a NanoDrop 1000 Spectrophotometer.

cDNA was synthesized from total RNA using the High Capacity cDNA Reverse Transcription Kit (Life Technologies) as per the manufacturer's protocol. TaqMan expression assays to determine RNA levels of *PIWIL1* (Hs01041737_m1), *PIWIL2* (Hs00216263_m1), *PIWIL3* (Hs00908825_m1), and *PIWIL4* (Hs00381509_m1) were supplied by Life Technologies. β-actin was used as housekeeping gene. RT-QPCR was performed in a total volume of 20 μl in the ABI Prism 7500 Real Time PCR System (Life Technologies). All samples for each gene were run in triplicate for 40 cycles using the following master mix and thermal cycler conditions: 10 μl of the TaqMan universal PCR master mix, 1 μl of the primers and probes, 2 μl of the cDNA and 7 μl of the RNAse-free water; about 2 min 50°C, 10 min 95°C, 15 s 95°C and 1 min 60°C. Fluorescent emission data were captured, and mRNA concentrations were quantified by using the critical threshold value and 2^−ΔΔCt^ method.

### Immunohistochemistry

Five-μm-thick transverse sections of formalin-fixed paraffin-embedded (FFPE) tissues were serially cut and mounted onto Dako Silanized Slides (Dako, Glostrup, Denmark). The immunohistochemical assay was performed as previously described [53] using rabbit polyclonal antibodies anti-human PIWIL1 (ab85125; Abcam, Cambridge, UK).

### Global methylation quantification

Global methylation was determined as previously described [54, 55] in total DNA of all samples by a specific ELISA assay (MethylFlash™ Methylated DNA Quantification Kit, Epigentek, Farmingdale, NY), which is capable of recognizing the DNA methylated fraction. This test uses a monoclonal antibody against 5-methylcytosine (5-mC) to obtain the percentage of 5-mC in total DNA. The procedure was performed according to manufacturer's protocol.

### *PIWIL1*-associated gene expression signature

Using arrays obtained from the Gene Expression Omnibus database (GSE10245), we examined the gene expression associated with patients who expressed *PIWIL1*. Raw files (.cel) were normalized using R statistical package (Institute for Statistics and Mathematics, Vienna, AU) and Bioconductor (www.bioconductor.org). The raw data was normalized using the rma method from the affy package (Bioconductor). After normalization, we obtained the expression data of *PIWIL1* (probe: 214868_at). The samples that had higher expression than the mean and standard deviation of the population were classified as *PIWIL1*-positive. A t-test based on permutations was used to identify genes differentially expressed between patients with high and low *PIWIL1* expression. The Gene Set Enrichment Analysis (GSEA v.2.1.0) program from “The Broad Institute” [56] was used to identify the enriched gene signatures associated with *PIWIL1* expression.

### Cell culture and treatment

Two NSCLC cell lines, H23 (American Type Culture Collection) and A549 (DSMZ - the German Resource Centre for Biological Material) were used. H23 and A549 cells were cultured in RPMI 1640 (Invitrogen) and DMEM (Invitrogen) respectively, containing 10% fetal calf serum (Invitrogen).

For methylation analysis, both cell lines (5 × 10^5^ cells) were plated 24 h prior to treatment. Cells were then treated daily with 1 μM/3 μM/10 μM 5-Aza-2-deoxycytidine (5-Aza-dC) during 6 days; on the 7th day, the cells were harvested and gene expression was assessed.

### Bisulfite sequencing

The methylation status of *PIWIL1* was studied by bisulfite sequencing. Following sodium bisulfite treatment using Methylamp DNA Modification Kit (Epigentek, Farmingdale, NY), the CpG island located in the 5′ region of *PIWIL1* gene (UCSC GRCh37/hg19: chr12:130822361–130822696) was PCR amplified using Forward 5′-GGGGTTTTTTTGGTTT-3′ and Reverse 5′-CACCTAACACCTCAACCTAACC-3′ primers. PCR products were subcloned using the TOPO TA Cloning kit (Invitrogen, Life Technologies) into pCR2.1-Topo TA Vector plasmid and 5 candidate plasmid clones were sequenced. The resulting sequences were analyzed using BISMA [57].

### Statistical analyses

The primary endpoints of the study were to assess time to relapse (TTR) and overall survival (OS), calculated from the time of surgical treatment to the date of relapse or death from any cause, respectively. TTR and OS were calculated using the Kaplan–Meier method and compared using the log-rank test. Optimal cut-off points of gene expression data for TTR were assessed by means of maximally selected log-rank statistics [24] using the Maxstat package (version 2.8.1; R statistical package) and confirmed by the Kaplan–Meier test. All variables with a *p*-value ≤ 0.1 in the univariate analysis were included in a Cox multivariate analysis in order to calculate the independent odds ratios (ORs) for TTR and OS. All statistical analyses were performed with PASW Statistics 18 (SPSS Inc., Chicago, IL, USA) and R version 2.13. Statistical significance was set at *p* ≤ 0.05.

## SUPPLEMENTARY TABLE AND FIGURE




