# Supplementary material for: The significance of PIWI family expression in human lung embryogenesis and non-small cell lung cancer
Source: Oncotarget. 2015 Jan 23;6(31):31544–56. doi: 10.18632/oncotarget.3003 (PMC4741623; doi:10.18632/oncotarget.3003)
Supplement: Supplementary file 1 [file oncotarget-06-31544-s001.pdf]

SUPPLEMENTARY TABLE AND FIGURE

Supplementary Table 1: Genes differentially expressed between *PIWIL1*-positive and *PIWIL1*-negative cases obtained in the *in silico* analysis. The median expression of each gene is shown, together with the raw and the adjusted *p*-value for the *t*-test based on permutations. Upregulated (in red) and downregulated (in green) genes in the *PIWIL1*-positive group are indicated

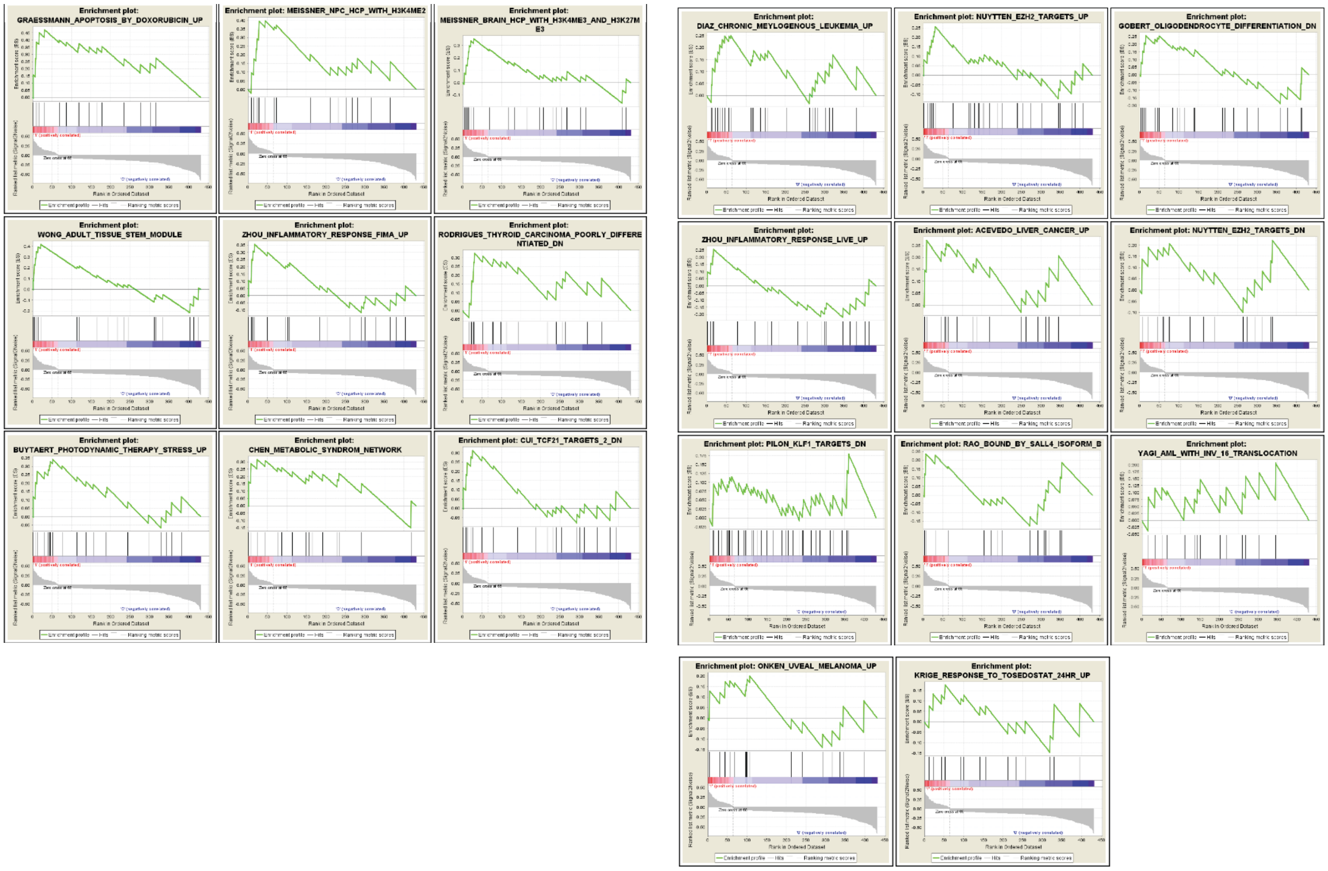

Supplementary Figure 1: Snapshot of enrichment results obtained after GSEA analysis performed using the "c2.all.v4.0.symbols.gmt" gene set database.
